# Evaluating the Effectiveness of Pubertal Preparedness Program in Terms of Knowledge and Attitude Regarding Pubertal Changes Among Pre-Adolescent Girls

**Published:** 2016-09

**Authors:** Manisha Rani, Poonam Sheoran, Yogesh Kumar, Navjyot Singh

**Affiliations:** 1 Department of Child Health Nursing, M.M. Institute of Nursing, Mullana, Ambala, India; 2 MM Institute of Nursing, Mullana, Ambala, India; 3 MM College of Nursing, Ambala, India; 4 Rama College of Nursing, Ghaziabad, India

**Keywords:** Adolescent, Biological Process, Female, Puberty, Sexual Maturation, Pubertal Preparedness Program, Questionnaire

## Abstract

**Objective:** To compare the knowledge and attitude regarding pubertal changes among pre – adolescent girls before and after the pubertal preparedness program (PPP) in experimental and comparison group.

**Materials and methods:** A Quasi experimental (non- equivalent comparison group pretest posttest) design was adopted with 104pre-adolescentgirls (52 in each experimental and comparison group) of age 12-14years, selected by purposive sampling from two different Government schools of Ambala District. Knowledge and attitude was assessed using structured knowledge questionnaire (KR-20 = 0.74) and 5 point likert scale (Cronbach’s alpha = 0.79) respectively. On the same day of pretest, PPP was administered and on 12^th^ day FAQs reinforcement session was held only for experimental group. After 28 days, posttest was taken.

**Results:** The computed t value of pretest of knowledge and attitude scores of pre-adolescent girls (1.97), (1.95) respectively in experimental and comparison group was found non-significant at 0.05 level of significance which shows that both group didn’t differ significantly in their knowledge and attitude before the administration of intervention. Findings of unpaired ‘t’ value of posttest knowledge and attitude scores of pre-adolescent girls (19.77), (17.17) respectively in experimental and comparison group were found significant at 0.05 level of significance, Thus knowledge and attitude of pre-adolescent girls were improved with PPP and FAQs session.

**Conclusion:** Pubertal preparedness program and FAQs reinforcement session are effective in enhancing knowledge and developing favorable attitude among pre-adolescent girls.

## Introduction

Adolescence is a period of transition between childhood and adulthood – a time of rapid physical, cognitive, social, and emotional maturation as the girls prepares for womanhood. The precise boundaries of adolescence are difficult to define, but this period is customarily viewed as beginning with the gradual appearance of secondary sexual characteristics at about 11 or 12 years of age and ending with cessation of body growth at 18 to 20 years. Adolescence which literally means, “to grow into maturity”. It involves three distinct sub phases: early adolescence (pre adolescence) (age 11 to 14 year), middle adolescence (ages 15 to 17 year), and late adolescence (ages 18 to 20 years) (1). Adolescents – defined by the United Nations as those between the ages of 10 and 19 – number 1.2 billion in 2010, forming 18 per cent of world population (2). Adolescent population in India has increased from 85 million in 1961 to 253 million in 2011 (in five decades) (3) and in Haryana percentage of adolescent’s population is approx.21% (4).

The most dramatic changes related to adolescence are the physical changes that occur as a part of pubertal process (5). Puberty includes maturational, hormonal, and growth process that occurs when the reproductive organs begin to function and the secondary sex characteristics develop (1). During puberty growth is disorganized confusing and rapid, compared to the relatively stable earlier period of childhood. When pubescent children are not informed of the changes that take place at puberty, it is traumatic to undergo these changes and may develop unfavorable attitudes towards these changes (6).

Studies have shown that there are still many misconceptions and misbelieves regarding issues related to sexuality and adolescence, which should be tackled comprehensively by imparting formal puberty and sex education at proper age (5). Various studies concluded that reproductive health is ignored and queries go unanswered (7). Adolescent possess some knowledge about reproductive health but still effective educational intervention is required to encourage more sensible and healthy behavior and results of a study shows health education sessions are very effective in increasing knowledge (8).

The response to physical changes of pubertal growth and development differs depending on the stage of development. Young adolescents become pre occupied with the rapid changes in their body and are interested in the anatomy, physiology and function of their sexual organs. 

For girls increase in weight, associated fat deposition and menstrual period may be distressing and frightening event. All teenagers, regardless of gender, are concerned with the question, “Am I normal”. To answer this question, they compare their body with the bodies of their peer and with images in the media. This leads to a great deal of uncertainty about their appearance and attractiveness. If an adolescent does not enter puberty at the same time as his or her peers, considerable inner conflicts may occur. So in this time when early adolescence experiences these physical, psychological and emotional changes, to deal these early adolescents requires the information regarding the bodily changes in order to prevent the problems like guilt and confusion. This is the time to seriously think about providing the right stimulus, role models and environment for adolescent girls, in order for them become assets for nation building. They have the potential; now is the time to provide them with the right way (1).

Results of a cross-sectional study conducted in 2011 to assess the knowledge regarding pubertal changes among 138 adolescent girls studying in Government Girl's School located in Darjeeling district, West Bengal shows that out of 138 students only 28.6% had knowledge of growth of pubic hair, 54.3% student faced difficulties related to adolescent period. 58.7% found necessary solution from their peers and only 39.1% discussed with parents. So, an educational intervention in order to provide accurate and authentic knowledge about reproductive health, related crucial issues is needed to enable them to make right decisions in life (9).

With this background, the study was aimed to assess and compare the knowledge and attitude of pre-adolescent girls in experimental and comparison group before and after the pubertal preparedness program.

## Materials and methods

This quantitative study was based onquasi experimental (nonequivalent comparison group pretest posttest) design to test and compare participants at two specified time points (initially before pubertal preparedness program and 28 days after that).

The study was conducted in two government schools of Ambala District Haryana, selected by convenience sampling and allotted to experimental and comparison groups by lottery method. Data was collected after obtaining clearance from the “institutional ethical committee”.

The study participants selected by purposive sampling technique comprised of 104pre-adolescentgirls (52 in each experimental and comparison group) of 12 -14 years age group studying in 7^th^ and 8^th^ class from two government schools (selected by convenience sampling) of Ambala District Haryana, and allotted to experimental and comparison groups by lottery method.

Ethical consideration was taken from the MM University institutional ethical committee (under the project number 375). Written informed assent was also obtained from all the participants before starting the study.

Knowledge and attitude was assessed before and 28 days after the pubertal preparedness program using a structured knowledge questionnaire and attitude scale. Areas of the multiple choice questions were reproductive organs, concept of puberty, secondary sexual characteristics, menstruation and emotional changes. 

A five point likert scale ranging strongly agree to strongly disagree containing 33 statements was used out of which 17 were positive statements and 16 were negative. The maximum score was 165 and minimum score was 33. Areas included were concept of puberty, secondary sexual characteristics, menstruation and emotional changes. Both were validated by 7 experts in the various nursing fields.

After obtaining the formal approval from the principals of two Government schools of Ambala district, Haryana, on first day pretest was conducted to assess the knowledge and attitude regarding pubertal changes in experimental group and on the same day pubertal preparedness program (PPP) was administered using audio visual aids. After that on 12^th^ day reinforcement session using FAQs was held to develop favorable attitude among pre-adolescent girls and posttest was conducted after 28 days of PPP. For comparison group on first day pretest and after28 days post test was conducted to assess the knowledge and attitude regarding pubertal changes without giving any intervention. Pubertal preparedness program (PPP) of duration 45-50 minutes was structured for enhancing knowledge and to develop favorable attitude regarding pubertal changes among pre-adolescent girls

The broad content outline included Introduction about the female reproductive system, Puberty and various stages of pubertal period, Physical changes – secondary sexual characteristics, menstruation and various emotional changes that occur during puberty.

Lecture cum discussion method was adopted for teaching.

Data were entered into Microsoft Excel 2007 and analyzed using SPSS 17.0. Categorical data are presented as mean (SD) or median based on the distribution of data. Statistical analysis was performed by using t test for continuous variables and chi square for categorical variables. 

A p value of 0.05 was considered significant.

## Results

Frequency, percentage distribution and chi square was computed to describe the sample characteristics of the sample and characteristics similarities of the sample in both experimental and comparison group. The baseline sample characteristics of the participants showed that in experimental group and comparison group more than half girls (53.8%), (61.5%) were of 7^th^ class and remaining (46.2%), (38.5%) were of 8^th^ class respectively. Among these 40.4% and 48.1 %were of 13 years age. Most of girls (78.8%), (73.1%) were Hindu respectively. In experimental group and comparison group both more than half girls (51.9%) (53.8%) were from joint families, and remaining were nuclear families. Majority of pre-adolescent girls (94.2%) of experimental group and 82.7% of comparison group were residing in rural area. In experimental group less than half of girls’ fathers (34.6%) were educated up to secondary and mothers, 38.5% were non literate and in comparison group 38.5% father and 36.5% mothers had education up to higher secondary. Majority of girls’ father (65.4%) in experimental group and 72% in comparison group were laborer and Mothers of majority of girls in experimental group (73.1%) and (84.6%) in comparison group were home maker. 100% girls in experimental group and 98.1% in comparison group had knowledge regarding puberty and for them source of information were parents books and elder siblings.

The computed chi square value for the sample characteristics of experimental and comparison groups’ girls were found to be non-significant at 0.05 level of significance so this revealed that girls in both groups were homogenous with regard to the selected sample characteristics before the administration of pubertal preparedness program.

In pretest of structured knowledge questionnaire majority of pre-adolescent girls in experimental group (82.7%) and in comparison group (94.2%) had below average knowledge and in posttest of experimental group (50%) girls had good and (44.24%) had very good knowledge whereas in comparison group, in posttest majority of girls (84.61%) had below average knowledge***.***

**Table 1:** Area Wise Mean, Mean Difference, Standard Deviation of Difference Standard Error of Mean Difference and ‘t’ Value of Pretest and Posttest Knowledge Score of Pre-Adolescent Girls in Experimental and Comparison Group

Group	Area	Pre test Mean	Post test Mean	Mean _D_	SD_D_	SE_MD_	t value
**Experimental ** **(n =52)**	**Reproductive organs**	**1.55**	**3.02**	**1.46**	**1.24**	**0.17**	**8.47** *
	**Concept of puberty**	**0.96**	**3.75**	**2.78**	**1.47**	**0.20**	**13.65** *
	**Secondary sexual characteristics**	**1.53**	**2.69**	**1.15**	**1.52**	**0.21**	**5.45** *
	**Menstruation**	**7.84**	**13.4**	**5.50**	**2.77**	**0.38**	**14.28** *
	**Emotional changes**	**2.32**	**3.23**	**0.90**	**1.12**	**0.15**	**5.79** *
**Comparison group** **(n =52)**	**Reproductive organs**	**1.21**	**1.44**	**0.23**	**1.30**	**0.18**	**1.272** ^NS^
	**Concept of puberty**	**0.98**	**0.82**	**0.15**	**1.19**	**0.16**	**0.929** ^ NS^
	**Secondary sexual characteristics**	**1.80**	**1.46**	**0.34**	**1.63**	**0.22**	**1.530** ^ NS^
	**Menstruation**	**6.63**	**7.55**	**0.92**	**3.28**	**0.45**	**2.028**
	**Emotional changes**	**2.26**	**2.42**	**0.15**	**1.85**	**0.25**	**0.599** ^ NS^

n = 104;

‘t’ (51) = 1.675 (p ≤ 0.05);

* significant; NS- non significant

In pretest of attitude scale, majority of pre-adolescent girls in experimental group (90.39%) and in comparison group all (100%) had moderately favorable attitude, whereas no one had favorable attitude and in posttest for experimental group (53.8%) had moderately favorable and (46.2%) had favorable attitude, whereas in comparison group most of (96.16%) had moderately favorable attitude and no one had favorable attitude.

The computed ‘t’ value of pretest knowledge and attitude scores of pre-adolescent girls (1.97) and (1.95) respectively was found to be statistically non-significant at 0.05 level of significance thus suggesting that both experimental and comparison groups does not differ in their knowledge and attitude significantly before the administration of intervention and for posttest findings of unpaired ‘t’ value of knowledge and attitude score of pre-adolescent girls (19.77), (17.17) respectively in experimental and comparison group were found significant at 0.05 level of significance, Thus knowledge and attitude of pre-adolescent girls were improved with PPP and FAQs session.

In order to determine the significance between pretest and posttest area wise ‘t’ value was computed and presented in table 1 and 2

The computed ‘t’ value for knowledge scores in experimental group was found to be statistically significant at 0.05 level of significance in all areas that were reproductive organs, concept of puberty, secondary sexual characteristics, menstruation and emotional changes but in comparison group computed ‘t’ values for all areas were found to be statistically non-significant at 0.05 level of significance except area of menstruation (2.028), this increase in area could be because of sensitization of pretest or some of them might had experienced menstruation in mean time.

**Table 2:** Area Wise Mean, Mean Difference, Standard Deviation of Difference Standard Error of Mean Difference and‘t’ Value of Pretest and Posttest Attitude Score of Pre-adolescent Girls in Experimental and Comparison Group

**Group **	**Areas**	**Pre test ** **Mean**	**Post test ** **Mean**	**Mean **_D_	**SD**_D_	**SE**_MD_	**t value**
Experimental (n = 52)	Concept of puberty	43.67	55.86	12.19	6.18	0.85	14.21*
	Menstruation	22.32	29.03	6.71	10.41	1.44	4.64*
	Secondary sexual characteristics	7.28	10.73	3.44	4.08	0.56	6.07*
	Emotional changes	21.67	27.19	5.51	4.88	0.67	8.14*
Comparison group (n = 52)	Concept of puberty	46.21	44.32	1.88	6.66	0.92	2.038*
	Secondary sexual characteristics	20.92	21.42	0.50	4.23	0.58	0.852^NS^
	Menstruation	8.51	7.61	0.90	3.24	0.45	2.006*
	Emotional changes	22.36	22.57	0.21	6.16	0.85	0.247^ NS^

n = 104;

‘t’ (51)= 1.675 (p ≤ 0.05);

* significant; NS- non significant

**Figure 1:**
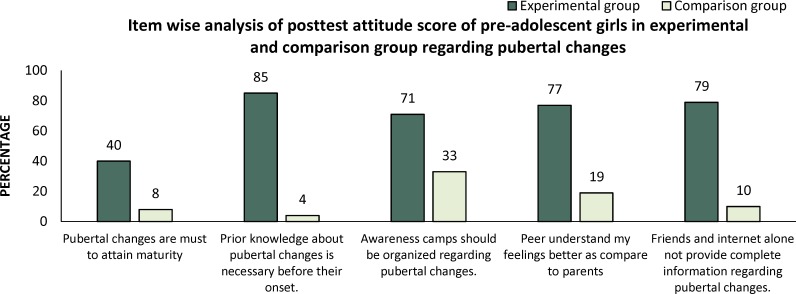
Item wise analysis of positive statements of post-test attitude score of pre-adolescent girls in experimental and comparison group regarding pubertal changes

The computed ‘t’ value for attitude scores in experimental group was found to be statistically significant at 0.05 level of significance in all areas that were concept of puberty, secondary sexual characteristics, menstruation and emotional changes but in comparison group computed ‘t’ value for all areas was found to be statistically non-significant at 0.05 level of significance in all areas except area of “concept of puberty” (2.038) and area of “menstruation” (2.006), this increase in area could be because of sensitization of pretest or some of them might had experienced menstruation in mean time.

**Figure 2:**
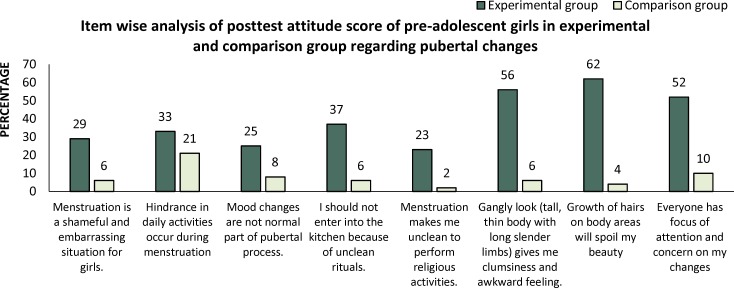
Item wise analysis of negative statements of post-test attitude score of pre-adolescent girls in experimental and comparison group regarding pubertal changes

Frequency and percentage distribution of item wise analysis positive and negative statements of posttest attitude score was calculated to evaluate the effectiveness of PPP on pubertal changes in terms of gain of attitude scores of pre-adolescent girls in both experimental and comparison group. Figure 1 and figure 2.

In positive statements highest score (5) was given to strongly agree whereas in negative statements highest score (5) was given to strongly disagree. 

The results of experimental and comparison group differs accordingly. In experimental group (who attend pubertal preparedness program) percentage was increased towards strongly agree in positive statements and towards strongly disagree in negative statements.

**Figure 3:**
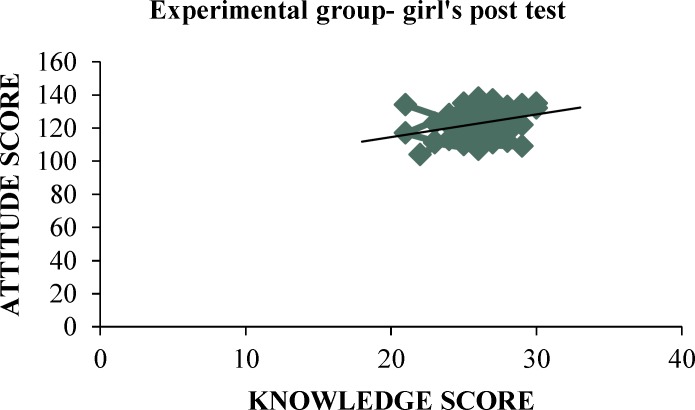
Scatter Plot Showing the Correlation between Knowledge and Attitude ScoresObtained

A significant low positive correlation was found between mean posttest knowledge and attitude scores of pre-adolescent girls regarding pubertal changes as evident by computed ‘r’ value of (0.32) as shown in figure 3. 

The findings of Anova/t values showing the association of level of post knowledge and attitude scores of pre-adolescent girls with educational status of mother (0.029), source of information (0.001) and (0.003) respectively were found to be significant at 0.05 level of significance, it denotes the association of this with knowledge and attitude scores. Friends were major source of information regarding pubertal changes.

## Discussion

The finding of study further revealed that 19,2% girls were aware about concepts of puberty, 38.75% girls have knowledge about reproductive organs and their functions, 30.6% girls were aware about secondary sexual characteristics and 46.4% girls, were aware about emotional changes. The study finding were inconsistent with finding of a cross sectional study conducted in block Beri, District Jhajjar (Haryana) regarding assessment of self-awareness of rural adolescent students regarding adolescent changes. Findings shows that 61.25 % girl were aware about physical changes in their bodies during puberty, 63.75 % girls, have knowledge about sexual development changes and total 23% were aware about emotional changes during puberty. Reasons for the inconsistent findings may be due to the difference in the settings (10).

The results of study revealed that (82.7%) girls had below average knowledge and (17.3%) had average knowledge and (10%) had unfavorable and (92%) had moderately favorable attitude. Similar findings were reported in a study conducted among intermediate school female students in Taif, Saudi Arabia, to assess knowledge and attitude regarding changes occurring during puberty. Findings shows that less than half of the female students (43.8%) had below average and slightly more than half (56.2%) had an above average level of knowledge and (38.9%) had unfavorable and (61.1%) had moderately favorable attitude towards pubertal changes (11).

In present study for most of girls’ source of information were parents (mothers) and friends, These findings were consistent with study held in Turkey on, matter of reproductive health, shows that girls mostly discuss their puberty symptoms with their mothers (82.8%) and friends (6.5%) (12).

## Conclusion

Pre- adolescent girls, who were exposed to PPP (Pubertal preparedness program), had significantly higher knowledge and favorable attitude than pre - adolescent girls who did not exposed to PPP. Therefore the study concluded that structured pubertal preparedness program and FAQs (frequently asked questions) reinforcement session was effective in terms of enhancing knowledge and developing favorable attitude of pre - adolescent girls regarding pubertal changes. The finding of present study has implication in various areas of nursing namely: nursing practice, nursing administration, community health nursing and mass media.

Regular health education program conducted by the nursing personnel in the community areas helps pre-adolescent girls to deal with pubertal changes.

Nursing administration could provide the necessary facilities like educational material, pamphlet, hording and opportunities etc. for nursing staff to equip themselves with knowledge related to pubertal changes. The school nurse has a crucial role in the seamless provision of comprehensive health services to adolescents. She serves in providing preventive services, early identification and interventions for adolescent reproductive and sexual health problems to foster good health.

The basic functions of media are surveillance, interpretation, linkage, transmission of values and entertainment. Media, therefore, plays a major role in shaping the attitudes, perception and beliefs of pre adolescents. It also has power to create and change stereotype. If mass media messages are delivered by strong role models, behavior change can be dramatic.
